# A Comparative Analysis of Distal Femur Plating and Intramedullary Nailing in the Management of Distal Femoral Fractures: A Retrospective Cross-Sectional Study

**DOI:** 10.7759/cureus.112032

**Published:** 2026-07-04

**Authors:** Raghu Shravan, Anil Kumar Prakash, Nagakumar JS, Hariprasad Seenappa

**Affiliations:** 1 Department of Orthopedics, Sri Devaraj Urs Academy of Higher Education and Research, Kolar, IND

**Keywords:** distal femoral fracture, fracture union, locking compression plate, orthopaedic trauma, orthopedic trauma, retrograde nailing

## Abstract

Background: Distal femoral fractures constitute a significant proportion of all femoral fractures and are increasingly common due to the aging population and high-energy trauma in younger individuals. Surgical management is preferred for most cases, with the two predominant modalities being distal femoral locking plates (DFLP) and retrograde intramedullary nailing (RIMN). The choice between plating and nailing remains controversial, with ongoing debate regarding optimal treatment strategies based on fracture characteristics, patient factors, and surgeon preference.

Objective: The primary objective of this study was to compare the time to radiographic fracture consolidation between patients treated with DFLP fixation and those treated with RIMN. Secondary objectives included comparison of functional outcomes at six months, as measured by the Knee Society Score (KSS), overall union rates, including delayed union and nonunion, and complication profiles, encompassing surgical site infection, implant failure, and postoperative malalignment.

Methods: A retrospective cross-sectional study was conducted between November 1, 2023, and October 31, 2025, including 46 samples. The key outcome measures included operative time, union rate, time to union, functional scores (KSS), infection rate, malalignment, and implant failure.

Results: Plating showed slightly higher nonunion and delayed union rates, especially in cases with poor soft tissue or comminution. Measured by KSS, both groups demonstrated comparable long-term functional outcomes. Plating had higher superficial infection rates due to wider exposure. RIMN showed higher malalignment, often valgus, due to technical limitations during reduction.

Conclusion: There is no definitive superiority between distal femur plating and nailing. Both yield excellent outcomes when applied judiciously. DFLP is preferred in intra-articular, osteoporotic, and comminuted fractures. RIMN is ideal for simple, extra-articular fractures and for younger patients. Surgeon familiarity and patient-specific factors remain pivotal in decision-making.

## Introduction

Distal femur fractures are among the more difficult injuries faced by orthopedic trauma surgeons. They are challenging due to the complex anatomy around the knee, the varied fracture patterns that can occur, and the high functional demands placed on patients' knees [1]. These fractures account for a significant proportion of all femur fractures and occur by two separate mechanisms. In younger patients, they are usually the result of high-energy trauma, including motor vehicle accidents or falls from heights. In older people, they arise from trivial falls wherein the bone succumbs to underlying osteoporosis [2].

Surgery is now the gold standard for the management of these fractures. Allowing these injuries to heal without surgery or to be managed in a cast often results in poor outcomes: bones heal in less-than-ideal positions, joints become stiff with prolonged immobilization, and patients endure lengthy recoveries that may never restore normal function [2]. On the other hand, operative treatment offers the opportunity to restore normal anatomy, stabilize the fracture, and mobilize patients early to optimize their strength and mobility [3].

In the last few decades, both implant technology and surgical thinking have changed a lot. Two fixation methods are mainly discussed in the literature on distal femur fractures. The first is known as a locking compression plate. This design employs screws that thread into the plate itself, with a fixed-angle construction. This presents stability for multifragment fracture patterns. Modern plating techniques also minimize disruption of the soft tissues around the bone and preserve some of the callus potential for healing [4].

A second option is the distal femoral nail, a type of retrograde intramedullary nail that can be introduced through the knee joint and will pass across the fracture site. This approach is fundamentally different. Instead of sitting on the outside like a plate, the nail works from within, acting as a load-sharing device. It passes through small incisions, disrupts very little soft tissue, and preserves the fracture hematoma. This biological preservation is believed to accelerate healing and reduce the risk of infection. Those who undergo nail treatment usually start moving earlier than those whose operation is performed with plates [4,5].

Despite considerable global experience with both implants, the literature has not reached a clear consensus on preference. The practice of nailing is faster, involves less blood loss, and allows early weight bearing. Others have shown that plates offer better control of complex fractures, especially those involving the knee joint itself, achieving similar overall complication rates. A large prospective observational study demonstrated that nails offered intraoperative and postoperative advantages along with faster fracture healing; however, at the two-year follow-up and beyond, they did not demonstrate superior functional outcomes compared to the alternative [1]. Another comparative analysis revealed that both implants achieved acceptable union rates; however, nails were associated with a higher incidence of anterior knee pain, whereas the technical difficulty of implant insertion varied according to the fracture configuration [4,5]. Population-based registry data further support comparable outcomes between the two implants [6].

Larger studies using propensity score matching have shown no significant differences in revision surgery rates or radiographic outcomes between nails and plates [7]. A multicenter randomized trial comparing locked plating to retrograde nailing similarly demonstrated no significant difference in functional outcomes at one year, emphasizing the role of patient selection [8]. The findings of these studies suggest that the choice of implant may be less important than other factors, particularly careful patient selection, the surgeon's proficiency, and comfort with the preferred fixation method. Biomechanical studies further complicate matters by showing that different constructs exhibit varying stiffness and load-sharing properties, which may influence healing as a function of fracture configuration [9]. A multicenter randomized trial has similarly reported that locking constructs, while mechanically robust, do not uniformly reduce reoperation rates compared with alternative fixation strategies [10].

With this mixed evidence base, a careful comparison between locking plates and retrograde nails is warranted. Having a grasp not only of the numbers but also of the specific scenarios to which each implant is ideally suited should help surgeons make the right decisions. It is not a matter of crowning one technique the winner but of providing surgeons with sufficient education to use the appropriate implant in the right patient to aid recovery and reduce complications [11].

The primary objective of this study was to compare time to radiographic fracture consolidation between patients treated with distal femoral locking plate (DFLP) fixation and retrograde intramedullary nailing (RIMN) for Association for the Study of Internal Fixation/Orthopaedic Trauma Association (AO/OTA) type 33 distal femur fractures. Secondary objectives were to compare 1) functional outcomes at six months as measured by the Knee Society Score (KSS); 2) overall union rates, including rates of delayed union and nonunion; and 3) complication profiles encompassing surgical site infection, implant failure, and postoperative malalignment.

## Materials and methods

Study design and reporting

This retrospective cross-sectional study was conducted at R.L. Jalappa Hospital (RLJH) and Research Center between November 1, 2023, and October 31, 2025. Reporting followed the Strengthening the Reporting of Observational Studies in Epidemiology guidelines. Of the patients initially identified, all 46 who met inclusion criteria completed the minimum six-month follow-up and were included in the final analysis. No patients were lost to follow-up during the study period. Missing data were not encountered for the primary outcome measures; however, minor gaps in secondary documentation were resolved by cross-referencing operative notes and inpatient records.

Ethical approval

Institutional Ethics Committee approval was obtained (IEC reference number: SDUAHER/R&D/CEC/SDUMC-PG/303/NF/-2025-26) in accordance with the Declaration of Helsinki. Due to the retrospective design, informed consent was waived.

Participants

Patients aged 18 years or older operated on at RLJH, attached to Sri Devaraj Urs Academy of Higher Education and Research, who underwent DFLP or RIMN. AO/OTA type 33-A, 33-B, or 33-C distal femur fractures were included with a minimum six-month clinical and radiographical follow-up. Implant selection was determined by the operating surgeon based on fracture morphology, soft-tissue status, and patient factors.

Inclusion and exclusion criteria

The following were the inclusion criteria: age ≥18 years; AO/OTA type 33-A, 33-B, or 33-C distal femur fracture; operative fixation with DFLP or RIMN; and minimum six-month clinical and radiographic follow-up. The exclusion criteria were as follows: pathological or periprosthetic fractures; Gustilo-Anderson grade IIIB/C open injuries; bilateral distal femur fractures; polytrauma requiring intensive care; preexisting ipsilateral knee disease or prior femoral surgery; and incomplete records precluding primary outcome evaluation.

Surgeon selection criteria and potential for selection bias

Implant selection was surgeon-directed, not randomized. Surgeons typically favor RIMN for simpler extra-articular fractures (AO/OTA 33-A) with an intact canal and adequate distal bone stock, and DFLP for fractures with intra-articular extension (33-B/C), metaphyseal comminution, or compromised soft tissues. Surgeon familiarity with a given implant further influences this decision. This preferential allocation introduces indication bias: as evidenced by the significant baseline imbalance in fracture-type distribution (33-A more prevalent in RIMN, p = 0.038), simpler fractures were systematically assigned to nailing. Since fracture complexity independently predicts union rate and infection risk, observed outcome differences between the groups may reflect preexisting case-mix differences rather than true implant-specific effects, and all between-group comparisons must be interpreted with this limitation in mind.

Surgical technique

All procedures were performed with the patient in the supine position under spinal anesthesia with fluoroscopic guidance. Three professor-level orthopedic surgeons participated in this study, each with at least 10 years of experience in orthopedic trauma surgery. For DFLP fixation, a lateral or minimally invasive approach was utilized based on fracture pattern and soft-tissue condition. A tourniquet was applied, and open reduction was performed to achieve anatomic alignment prior to plate fixation. For RIMN, a closed reduction technique was employed, with the nail introduced through a standard retrograde entry point under fluoroscopic control. While professor-level surgeons participated in this study, all procedures were performed or directly supervised within a single institutional unit with a standardized operative protocol. Variability in surgical technique across cases represents an inherent limitation of the retrospective design and is acknowledged accordingly.

Postoperative protocol

All patients were maintained on strict non-weight-bearing status for the first four weeks postoperatively. At six weeks, progression to partial or toe-touch weight-bearing was permitted based on clinical and radiographic evidence of early fracture consolidation. Patients treated with RIMN were advanced to full weight-bearing at three months, while those treated with DFLP achieved full weight-bearing at six months. Physiotherapy was initiated immediately postoperatively for all patients, incorporating dynamic exercises and quadriceps strengthening to preserve joint mobility and prevent muscle atrophy. Follow-up occurred at six weeks, three months, and six months, with clinical and radiographic assessments at each visit.

Outcome measures

The primary outcome measure was functional recovery assessed using the KSS at final follow-up. The KSS is a validated clinician-reported instrument comprising two distinct domains: a knee score (evaluating pain, stability, and range of motion) and a functional score (evaluating walking distance and stair climbing ability) [12]. Radiographic union was defined as bridging callus across at least three of four cortices on both anteroposterior and lateral plain radiographs, in conjunction with clinical evidence of fracture consolidation, including absence of pain at the fracture site on weight bearing. Delayed union was defined as failure to achieve union by 20 weeks, and nonunion as failure to achieve union by 26 weeks with no radiographic evidence of progression.

Statistical analysis

Data were entered into the Statistical Package for the Social Sciences, version 26 (IBM Corp., Armonk, NY). Descriptive statistics are reported as mean ± standard deviation for continuous variables and as frequencies with percentages for categorical variables, with normality assessed using the Shapiro-Wilk test. Multivariable logistic and linear regression models were planned to adjust for potential confounders including age, fracture classification, and comorbidities; however, given the limited sample size of 46 patients, adequately powered adjusted analyses could not be performed without risk of model overfitting, and unadjusted comparisons are therefore presented. Furthermore, no formal inferential statistical testing was performed for complication outcomes, as the absolute event numbers were too small to satisfy the minimum cell frequency assumptions required for chi-square analysis or regression-based modeling. A formal power calculation was not performed prospectively, and this study is acknowledged to be underpowered for definitive comparative analysis. Any numerical differences reported between the groups, as well as residual confounding, are acknowledged as limitations; the findings should therefore be regarded as hypothesis-generating observations and preliminary data to inform the design of adequately powered future studies.

## Results

Demographic and baseline characteristics

Both groups had comparable mean ages in the late 30s (38.7 ± 10.2 years for DFLP vs. 39.2 ± 9.8 years for RIMN, p = 0.865), with well-matched age ranges (DFLP: 22-61 years; RIMN: 23-60 years), reflecting a predominantly younger, higher energy trauma cohort. Sex distribution was comparable (DFLP male:female 15:8 vs. RIMN 14:9, p = 0.765), as was laterality (right:left 12:11 in both groups, p = 1.000). Fracture type distribution, however, revealed a statistically significant imbalance. Type 33-A fractures, representing the simplest extra-articular morphology, were significantly more prevalent in the RIMN group (34.8% vs. 13.0%, p = 0.038). Type 33-B fractures were the most common subtype in both groups and were evenly distributed (DFLP 52.2% vs. RIMN 43.5%, p = 0.556). Type 33-C fractures, representing the most complex intra-articular patterns, were more frequent in the DFLP group (34.8% vs. 21.7%), though this difference did not reach statistical significance (p = 0.219). This imbalance in baseline fracture complexity is the most important confounding variable in this study and must be considered when interpreting all subsequent outcome comparisons, as simpler fractures in the RIMN group may independently account for some of the observed differences in union time and infection rate (Table 1).

**Table 1:** Demographic characteristics and fracture type distribution of study population DFLP: distal femoral locking plate; RIMN: retrograde intramedullary nailing; AO/OTA: Association for the Study of Internal Fixation/Orthopaedic Trauma Association

Characteristic	DFLP (n = 23)	RIMN (n = 23)	p value	Interpretation
Mean age (years)	38.7 ± 10.2	39.2 ± 9.8	0.865	Not significant (p > 0.05)
Age range (years)	22-61	23-60	-	Well-matched distribution
Male:female	15:08	14:09	0.765	Not significant (p > 0.05)
Right:left	12:11	12:11	1	Perfectly matched
Fracture type				
AO/OTA 33-A, n (%)	3 (13.0%)	8 (34.8%)	0.038	Significant (p < 0.05)
AO/OTA 33-B, n (%)	12 (52.2%)	10 (43.5%)	0.556	Not significant (p > 0.05)
AO/OTA 33-C, n (%)	8 (34.8%)	5 (21.7%)	0.219	Not significant (p > 0.05)

Union rate and time

RIMN was associated with earlier radiographic and clinical evidence of fracture consolidation by approximately 15 days on average (p = 0.025). The narrower standard deviation in the RIMN group (±2.8 vs. ±3.5) suggests more predictable healing. While delayed union (13.0% vs. 4.3%, p = 0.515) and nonunion (8.7% vs. 4.3%, p = 0.715) rates were numerically higher in the DFLP group, neither difference reached statistical significance, and these findings should be regarded as descriptive only given the small event numbers (Table 2).

**Table 2:** Union time, delayed union, and nonunion rate of study population DFLP: distal femoral locking plate; RIMN: retrograde intramedullary nailing

Parameter	DFLP (n = 23)	RIMN (n = 23)	p value	Difference (RIMN vs. DFLP)
Mean union time (weeks)	18.2 ± 3.5	16.0 ± 2.8	0.025	-2.2 weeks (faster)
Delayed union	3 (13.0%)	1 (4.3%)	0.515	-8.7% (lower risk)
Nonunion	2 (8.7%)	1 (4.3%)	0.715	-4.4% (lower risk)

Functional outcome

Both groups achieved excellent or good functional outcomes in 82.6% of patients, suggesting that either technique can be successful in appropriately selected cases. While functional outcomes appeared comparable between the two groups with no statistically significant difference, this finding should be interpreted with caution given the limited sample size. The study is likely underpowered to detect potentially meaningful differences in functional outcomes, and the absence of statistical significance should not be misconstrued as confirmation of equivalence between the two techniques (Table 3).

**Table 3:** Comparing the functional outcomes between both groups using the Knee Society Score DFLP: distal femoral locking plate; RIMN: retrograde intramedullary nailing

Functional score	DFLP (n = 23)	RIMN (n = 23)	p value	Difference (RIMN vs. DFLP)
Excellent-good	19 (82.6%)	19 (82.6%)	1	0%
Fair-poor	4 (17.4%)	4 (17.4%)	1	0%

Complications

Neither implant demonstrated uniform superiority. The data suggest a trend toward higher malalignment rates with RIMN and higher infection rates with DFLP. However, infections that were higher in DFLP almost always require reoperation (debridement, antibiotics, and possible implant removal). The data confirm that the tradeoff is primarily between infection (DFLP risk) and malalignment (RIMN risk), with no discernible difference in functional outcomes between the two implants (Table 4).

**Table 4:** Comparison of complications between the groups DFLP: distal femoral locking plate; RIMN: retrograde intramedullary nailing

Complication	DFLP (n = 23)	RIMN (n = 23)	p value	Relative risk (DFLP/RIMN)
Infection	2 (8.7%)	1 (4.3%)	0.551	2.00×
Malalignment	1 (4.3%)	2 (8.7%)	0.319	0.50×
Implant failure	1 (4.3%)	1 (4.3%)	1.000	1.00×
Reoperation	1 (4.3%)	1 (4.3%)	1.000	1.00×

## Discussion

The present study was undertaken to evaluate the comparative effectiveness of DFLP and retrograde intramedullary nails in the management of distal femur fractures in adult patients. Baseline demographic characteristics confirmed that the two groups were well matched for age (38.7 ± 10.2 vs. 39.2 ± 9.8 years, p = 0.865), sex distribution (male:female 15:8 vs. 14:9, p = 0.765), and laterality (right:left 12:11 vs. 12:11, p = 1.000). However, fracture type distribution revealed a statistically significant imbalance that is central to the interpretation of all outcome data. Type 33-A fractures, representing the simplest extra-articular morphology, were significantly more prevalent in the RIMN group (34.8% vs. 13.0%, p = 0.038). Type 33-B fractures, the most common subtype overall, were comparably distributed (52.2% vs. 43.5%, p = 0.556). Type 33-C fractures, representing the most complex intra-articular patterns, were more frequent in the DFLP group (34.8% vs. 21.7%, p = 0.219). This imbalance directly reflects the surgeon-directed nature of implant selection, in which nailing was preferentially used for simpler fractures and plating for more complex ones. As a result, the RIMN group carried an inherently more favorable fracture complexity profile, which must be considered as the primary confounding variable when interpreting all subsequent outcome comparisons. Observed advantages in union time and infection rate in the RIMN group may therefore reflect, at least in part, the simpler fracture morphology in that cohort rather than a true implant-specific benefit.

**Figure 1:**
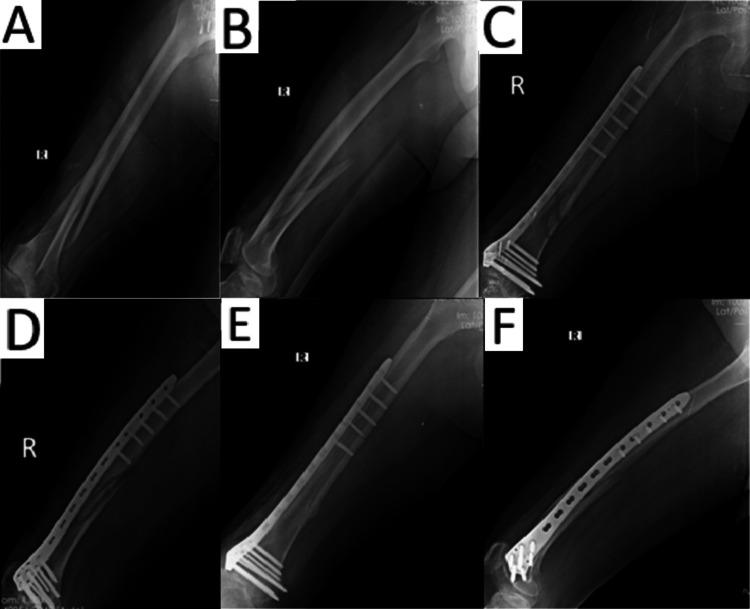
Pre-op (A) anteroposterior and (B) lateral views of the right femur. Post-op (C) anteroposterior and (D) lateral views of the right femur treated with distal femur plating. Six-month follow-up (E) anteroposterior and (F) lateral views of the right femur treated with distal femur plating

**Figure 2:**
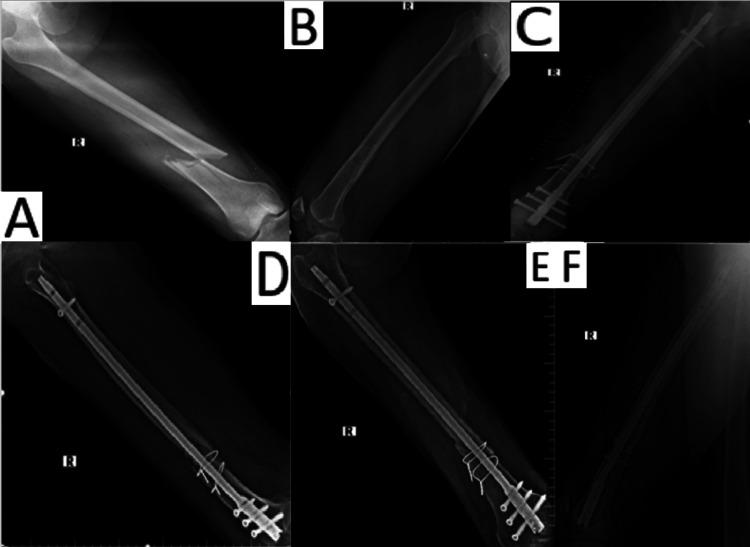
Pre-op (A) anteroposterior and (B) lateral views of the right femur. Post-op (C) anteroposterior and (D) lateral views of the right femur treated with retrograde intramedullary nailing. Six-month follow-up (E) anteroposterior and (F) lateral views of the right femur treated with retrograde intramedullary nailing

An observed trend toward earlier radiographic consolidation in the nailing group and narrower variability in healing times was noted (Figures 1, 2). However, this difference did not translate into any detectable functional advantage, and given the significant baseline imbalance in fracture complexity, with simpler fractures overrepresented in the RIMN group, it is not possible to attribute this finding to an implant-specific biological effect. The literature provides a plausible biological rationale for faster union with intramedullary devices, including load-sharing mechanics and preservation of the fracture hematoma [4,5], but this study cannot confirm that mechanism as the explanation for the observed difference. The finding should therefore be treated as an association requiring prospective confirmation rather than evidence that nailing accelerates healing in this patient population [13,14].

Functional outcomes, as measured by the KSS, were identical between the two groups, with both achieving the same proportion of excellent and good results. While union and complication data may invite further inference, the functional outcome scores do not support any meaningful difference between the two implants in this study [1]. Patients who heal more rapidly and with fewer complications are better positioned to engage in early rehabilitation, preserve joint mobility, and avoid the muscle atrophy that accompanies prolonged immobilization [5]. Both techniques performed comparably in most cases, reinforcing the principle that either implant can yield satisfactory results when used in appropriate clinical contexts [1,11]. Any suggestion of a functional advantage favoring one implant over the other is not substantiated by the present data and should not be overstated.

The complication profiles of the two implants reveal a distinct tradeoff that is central to surgical decision-making. Infection rates were markedly higher in the plating group, approaching double that of the nailing group. This disparity is readily explicable by the differing surgical approaches. Plate fixation requires larger incisions, greater soft-tissue dissection, and more extensive hardware exposure, all of which increase the risk of bacterial colonization and surgical site infection [4,7]. In contrast, the percutaneous nature of nailing minimizes soft-tissue trauma and preserves the integrity of the local biological environment, thereby reducing the risk of infection [5]. Contemporary consensus definitions classify these as fracture-related infections, which uniformly require aggressive surgical and antibiotic management [15].

**Figure 3:**
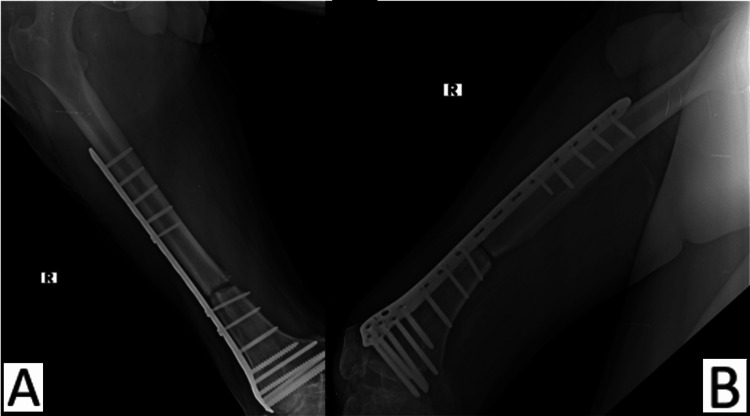
(A) Anteroposterior and (B) lateral X-ray views of distal femur plating showing nonunion (managed by exchange plating with bone graft application)

**Figure 4:**
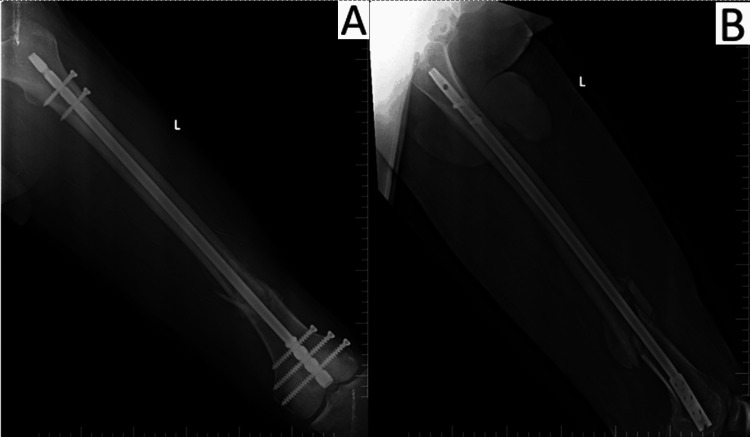
(A) Anteroposterior and (B) lateral X-ray views of retrograde intramedullary nailing showing nonunion (managed using bone marrow insertion)

Conversely, malalignment emerged as a significant drawback of intramedullary nailing. The nailing group exhibited a malalignment rate approximately twice that of the plating group (Figures 3, 4). This finding is consistent with the technical limitations of nailing, particularly in metaphyseal fractures, where the discrepancy between nail diameter and intramedullary canal width can permit angular deformity [4,9]. Plating, by virtue of allowing direct visualization and anatomic reduction, affords superior control over fracture alignment [7]. However, not all malalignments are clinically significant. Minor degrees of angulation may be well-tolerated functionally, whereas infections almost invariably mandate surgical intervention. This distinction likely explains why the reoperation rate, while higher in the DFLP group, did not differ to a statistically significant degree. Implant failure rates were virtually identical between groups, indicating that both devices provide adequate mechanical stability when osseous healing proceeds uneventfully [9].

Taken together, these findings suggest rather than confirm the absence of a universally superior implant, and all observed differences should be interpreted as associations within the constraints of a retrospective, underpowered study. Implant selection should remain guided by fracture morphology, soft-tissue status, and patient-specific factors [11,16]. The observed trend toward higher infection rates with plating and higher malalignment rates with nailing may inform clinical decision-making, but neither finding reached a level of evidence sufficient to support categorical recommendations [5,7]. The principal tradeoff between infection and alignment risk remains a clinically plausible framework, though its confirmation requires prospective validation in adequately powered studies [9].

The present study adds to the comparative literature on distal femur fixation in three respects. First, it provides single-institution data from a low-to-middle-income country surgical setting, a context underrepresented in the existing literature, which is dominated by high-volume North American and European registries whose findings may not be directly transferable to resource-limited environments. Second, by operating within a standardized protocol under a defined cohort of experienced surgeons, this study reduces the intersurgeon variability that commonly confounds retrospective multicenter comparisons. Third, and perhaps most importantly, it explicitly documents the baseline imbalance in fracture complexity between the two implant groups, an underreported confound in nonrandomized implant studies, and demonstrates that preferential assignment of simpler fractures to RIMN and more complex patterns to DFLP can independently influence outcome data. The observation that functional outcomes were equivalent despite differing complication profiles further reinforces the view that implant selection is secondary to fracture morphology, soft-tissue status, and surgeon expertise. These hypothesis-generating findings may serve as preliminary data to inform the design of adequately powered prospective trials in comparable patient populations.

The most significant limitation of this study is its sample size. With only 23 patients per group, the study is substantially underpowered to detect clinically meaningful differences between the two implants, and the absolute number of complication events in each category is too small to permit reliable bivariate analysis or multivariable adjustment for confounders. The absence of a prospective power calculation further compounds this limitation, and future studies should be designed with formal a priori sample size calculations targeting several hundred patients per group. Additionally, the nonrandomized design introduces selection bias, as implant choice was surgeon-directed rather than randomly allocated, raising the possibility that more complex fractures were systematically assigned to one treatment group. The analysis also did not account for fracture severity using established classification systems such as AO or Gustilo-Anderson, limiting the strength of causal inference that can be drawn from these findings. All comparisons should therefore be interpreted descriptively, and future studies incorporating detailed fracture characterization and adequate sample sizes would provide greater clarity regarding the influence of injury pattern on implant performance. A further and important limitation is the generalizability of these findings. With both groups having mean ages firmly in the late 30s and a predominance of high-energy injury mechanisms, this cohort is not representative of the geriatric distal femur fracture population. In many centers, particularly in the United States, patients over 50 years of age comprise the majority of distal femur fracture cases, and these patients present fundamentally different challenges, including osteoporotic bone quality, higher comorbidity burden, and distinct rehabilitation trajectories. The findings of this study should not be extrapolated to elderly or low-energy fracture populations, for whom dedicated prospective studies are needed and represent a significant gap in the current evidence base.

## Conclusions

This study provides preliminary observational data suggesting that both DFLPs and retrograde intramedullary nails are associated with comparable functional outcomes in adult distal femur fractures. Intramedullary nailing was associated with earlier radiographic consolidation and lower infection rates in this cohort, while plating was associated with lower malalignment rates. Given the retrospective design, small sample size, and significant baseline imbalance in fracture complexity between groups, these observations reflect associations rather than causal relationships and should not be interpreted as definitive evidence of implant-specific effects.

The key clinical message is that neither implant demonstrated superiority in functional outcomes, and this study does not provide sufficient evidence to recommend one implant over the other. Implant selection should remain individualized, guided by fracture morphology, soft-tissue condition, and surgeon expertise, with awareness of the observed tradeoff between infection risk with plating and malalignment risk with nailing. These findings are best regarded as preliminary and hypothesis-generating, and prospective randomized studies with adequate sample sizes and prespecified fracture stratification are needed before definitive practice recommendations can be made.
